# Large dynamic range Shack-Hartmann wavefront sensing based on a graph-theoretic computational model

**DOI:** 10.1038/s41377-026-02273-x

**Published:** 2026-04-15

**Authors:** Lintong Du, Rui Xu, Shuxin Liu, Rongjun Shao, Lin Li, Yuhang Zhang, Ziqiang Li, Yuan Qu, Dapeng Tian, Jiamiao Yang

**Affiliations:** 1 School of Automation and Intelligent Sensing, Shanghai Jiao Tong University, Shanghai, 200240 China; 2 State Key Laboratory of Submarine Geoscience, Shanghai Jiao Tong University, Shanghai, 200240 China; 3 State Key Laboratory of Dynamic Optical Imaging and Measurement, Changchun Institute of Optics, Fine Mechanics and Physics, Chinese Academy of Sciences, Changchun, 130033 China; 4 University of Chinese Academy of Sciences, Beijing, 100049 China; 5 Institute of Medical Robotics, Shanghai Jiao Tong University, Shanghai, 200240 China

**Keywords:** Imaging and sensing, Optical sensors

## Abstract

The Shack-Hartmann wavefront sensor (SHWS) is a widely used non-interferometric wavefront measurement technique. However, for high-slope wavefronts, spot crosstalk and asymmetric distortion cause severe matching ambiguity and centroiding errors. This creates an inherent conflict between dynamic range and reconstruction accuracy. To address this, a graph-theoretic computational model named G-SHWS is proposed. By minimizing the global pairing cost of a bipartite graph constructed between fitted and actual spots, G-SHWS drives the fitted distribution to approximate the true distribution and maps the subaperture attribution of the fitted spots to the actual spots, achieving precise spot-subaperture matching under severe aliasing. Furthermore, incorporating a Graph Attention Network (GAT) embedded with SHWS matching topology, the model utilizes a graph structure to explicitly encode the matching relationships obtained from the matching process, and combines the spatial features and intensity morphology of spots to achieve high-precision reconstruction of strongly distorted wavefronts, effectively circumventing the inherent centroiding errors under large aberrations. Experimental results demonstrate that G-SHWS extends the measurable range of SHWS to 21 times the conventional limit while maintaining a reconstruction error of less than $0.05{\rm{\lambda }}$, and remains robust under severe spot loss. These advancements significantly enhance the SHWS’s ability to measure complex aberrations, providing a reliable computational framework for large dynamic range wavefront sensing.

## Abstract (web summary)

This work proposes a graph-theoretic computational framework for large-dynamic-range Shack-Hartmann wavefront sensing, significantly extending its measurable range and ensuring robustness under spot loss.

## Graphical abstract

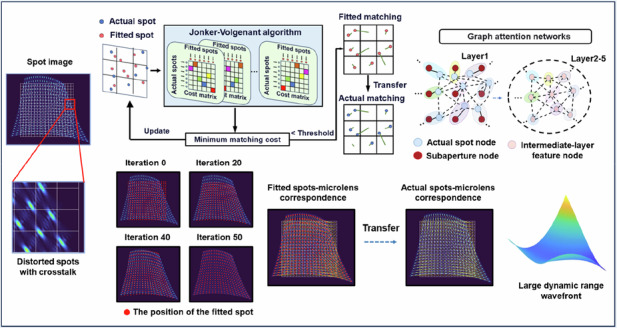

## Introduction

**Fig. 1:**
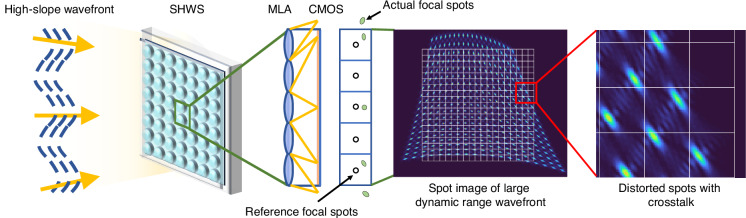
High-slope wavefront-induced spot crosstalk and intensity distribution distortion. Under high-slope wavefront input, the spots of the SHWS suffer from severe crosstalk and asymmetric distortion, leading to spot-subaperture mismatching and centroiding errors in conventional SHWS

As a representative non-interferometric wavefront sensing technique, the Shack–Hartmann Wavefront Sensor (SHWS) is widely employed in telescope observation^1,2^, biomedical imaging^3,4^, laser communication^5,6^, and quantum optics^7,8^. The system comprises a microlens array (MLA) and a camera, where the MLA partitions the incident wavefront into an array of subapertures. In each subaperture, the displacement of the focal spot is proportional to the local wavefront slope. By calculating the centroid shift of the spot relative to its reference position, the local phase gradient is obtained, and the full wavefront is subsequently reconstructed by fitting these gradients with Zernike polynomials^9,10^. However, as shown in Fig. 1, when the local wavefront slope becomes large, focal spots shift beyond their corresponding subaperture boundaries and overlap with neighboring regions, which causes spot-subaperture mismatching. Concurrently, spots with large displacements exhibit significant morphological distortion. In this scenario, centroid localization accuracy degrades severely, and the single centroid displacement becomes insufficient to accurately characterize the complex local wavefront information^11^. Collectively, these factors strictly restrict the measurable spot displacement of the SHWS within the physical boundaries of individual subapertures. This physical constraint limits the measurable wavefront slope range of the SHWS, which is referred to as the dynamic range^12^. Consequently, the limited dynamic range of the SHWS fundamentally restricts its capability in sensing high-slope complex wavefronts^13^.

Current research on expanding the dynamic range can be categorized into hardware improvements and algorithmic enhancements. In hardware schemes, reducing the microlens focal length is a straightforward approach but severely sacrifices measurement sensitivity^14^. Strategies employing masks or pinholes to occlude adjacent apertures effectively suppress crosstalk but require multiple scans, which significantly increases measurement time^15,16^. Furthermore, replacing the MLA with complex optical elements^4,17–19^ can partially enhance the dynamic range of SHWS, but it compromises system simplicity and cost-effectiveness, and faces challenges of limited field of view and low light efficiency stemming from device fabrication constraints^20^. In contrast, algorithmic methods offer greater flexibility and applicability without hardware constraints. Current approaches primarily rely on extrapolation—leveraging correlations among neighboring subapertures to propagate known local matches across the full field via greedy strategies such as unwrapping^21^ and iterative extrapolation^22–24^. These methods are effective within small dynamic ranges. However, when increasing wavefront slopes disrupt the spot topology, reliable local priors become unattainable and local continuity is broken, causing these methods to accumulate errors and ultimately fail in global matching. Some studies have proposed search methods based on the nearest neighbor principle^25^ and adjacent frame constraints^26^ to avoid the reliance on local matching priors and structural continuity, thereby extending the dynamic range to a certain extent. However, their search processes are still based on point-to-point matching via single-spot distance minimization without considering the global correlation of spot distribution, making the search prone to falling into local optima in the presence of severe spot crosstalk. Additionally, a fundamental bottleneck persists: these methods rely on centroiding for slope estimation. Even with correct matching, centroid positioning errors caused by spot distortion still lead to low reconstruction accuracy for large dynamic range wavefronts. Recent research has explored using Convolutional Neural Networks (CNNs) to directly achieve end-to-end “spot-to-phase” mapping^27–32^, but the sparsity of spot information in SHWS images and the difficulty for CNNs to explicitly model the spot-subaperture matching relationship limit their effectiveness in large dynamic range wavefront reconstruction. Therefore, achieving high-precision wavefront reconstruction under a large dynamic range remains a challenging task.

To address these challenges, this paper proposes G-SHWS, a graph-theoretic computational model designed to resolve the conflict between dynamic range and accuracy. G-SHWS adopts a global optimization concept based on graph theory. By optimizing the wavefront to minimize the global pairing cost of a bipartite graph formed between fitted and actual spots, it drives the fitted distribution to approximate the true distribution, thereby mapping the subaperture attribution of the fitted spots to the actual spots to achieve globally optimal spot-subaperture matching with high precision under severe aliasing. Furthermore, incorporating a matching topology-aware Graph Attention Network (GAT), the model explicitly encodes the spot-subaperture matching relationships obtained from the aforementioned matching process using a graph structure, and fuses spot spatial features with intensity morphology to achieve high-precision reconstruction of strongly distorted wavefronts, fundamentally avoiding the positioning errors inherent in conventional centroiding under distortion. G-SHWS achieves high-precision wavefront reconstruction over an extremely large dynamic range while maintaining robustness even under severe spot loss. This significantly extends the measurement capability of the SHWS, providing an effective computational solution for large dynamic range wavefront sensing.

## Results

### Principle and characterization of G-SHWS

This paper proposes G-SHWS, a graph-theoretic computational model designed to overcome the dynamic range limitations of Shack-Hartmann wavefront sensors. G-SHWS comprises two core components. First, to resolve matching ambiguities caused by crosstalk, ensuring robustness under severe spot aliasing and partial spot loss, G-SHWS employs a bipartite graph global combinatorial optimization strategy to reconstruct the global spot-subaperture matching relationship. Second, to transcend the representation limits of single-vector centroiding under large aberrations, G-SHWS constructs a Graph Attention Network that explicitly embeds matching topology. By fusing the global matching relationships with spot spatial features and intensity distribution morphology into high-order wavefront features, the network achieves high-precision reconstruction under a large dynamic range.

**Fig. 2:**
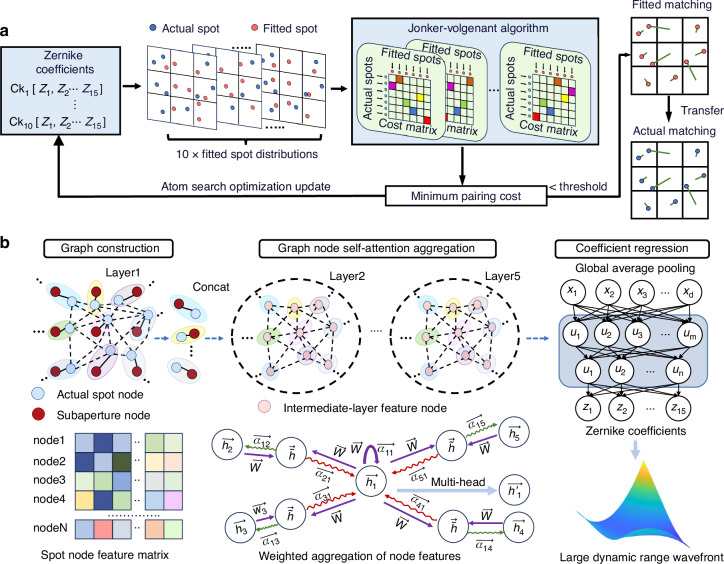
Overview of the G-SHWS framework. **a** Establishment of global matching relationship. Minimizing the global pairing cost of the bipartite graph constructed between fitted and actual spots drives the fitted distribution to approximate the actual one, mapping the sub-aperture attribution of the fitted spots to the actual spots. **b** Wavefront reconstruction using GAT. Actual spots and reference spots (sub-apertures) are abstracted as nodes with feature vectors, with edges representing matching relationships and spatial adjacencies. Matched nodes are concatenated to form the input feature $h$. $h$ is mapped via weight matrix $W$ and aggregates neighbor node features using attention coefficients ${{\rm{\alpha }}}_{{ij}}$ for weighted updates. After multi-layer stacking, Zernike coefficients $\left[{Z}_{1},{Z}_{2},\cdots ,{Z}_{15}\right]$ are regressed via fully connected layers

**Fig. 3:**
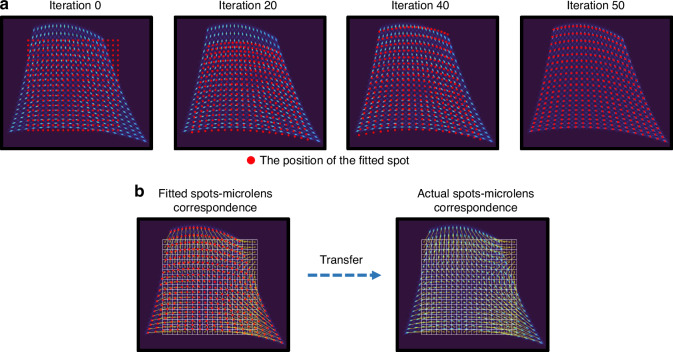
Iterative convergence of the global matching optimization process. **a** The fitted spot distribution progressively approximates the actual spot distribution through iterative optimization, ultimately converging to geometric alignment with the actual distribution. **b** Once the global matching relationship is established, the subaperture attribution of the fitted spots is transferred to their corresponding actual spots, achieving unambiguous spot-subaperture matching. Yellow lines represent the matching relationships

To address spot-subaperture mismatching caused by spot crosstalk under large dynamic range input, as illustrated in Fig. 2a, we reformulate the spot-subaperture matching problem as a global combinatorial optimization task. We treat the actual detected spot set and the fitted spot set (numerically generated from Zernike polynomials) as two independent node sets in a weighted bipartite graph^33^. The core mechanism operates as an iterative “distribution approximation” process (see Materials and methods: Global matching strategy). Specifically, the system constructs a cost matrix based on the Euclidean distances between every pair of actual and fitted spots. By employing the Jonker-Volgenant algorithm^34^ to solve for the Minimum Weight Perfect Matching (MWPM), we obtain the optimal pairing scheme between the actual and fitted spot node sets that minimizes the total distance cost. This minimum cost essentially quantifies the geometric discrepancy between the two distributions and serves as the cost function for the Atom Search Optimization (ASO) algorithm^35^. ASO iteratively explores the 15-dimensional Zernike coefficient space to dynamically adjust wavefront parameters to minimize this pairing cost. As illustrated by the convergence process in Fig. 3, this optimization drives the fitted spot distribution to progressively approximate the actual spot distribution. Once the global pairing cost converges to a minimum, implying that the fitted distribution aligns geometrically with the actual one, the system unambiguously transfers the known subaperture attributions of the fitted spots to their paired actual counterparts, thereby recovering the globally unique and correct spot-subaperture matching relationship. Furthermore, to ensure robustness against spot loss, we introduce high-cost virtual nodes into the cost matrix. This mechanism acts as a penalty filter, enabling the solver to automatically identify and discard unpaired nodes resulting from spot loss, thus ensuring the recovery of the correct matching relationship even under conditions of severe spot absence.

Following the recovery of the global spot-subaperture matching relationship, to address the failure of conventional centroiding methods caused by asymmetric spot broadening and stretching under large aberrations, we propose a Graph Attention Network (GAT) incorporating the SHWS matching topology to achieve high-precision wavefront parameter regression (see Materials and methods: GAT-based reconstruction network). Unlike conventional Convolutional Neural Networks (CNNs), which struggle to model matching relationships, the proposed GAT explicitly encodes the spot-subaperture matching topology into the network structure. The network abstracts actual spots and their corresponding subapertures as graph nodes. To fully exploit the high-order aberration information inherent in spot intensity morphology, the feature vector of each spot node comprises not only spatial extreme coordinates (boundary information) but also normalized second-order central moments and Hu invariant moments as morphological descriptors. These feature vectors are fused with the position encodings of the corresponding subapertures to constitute the input state of each node. Meanwhile, for subapertures with missing spots, proxy spots are generated using the converged estimated wavefront from the matching stage, which are also abstracted as spot nodes to preserve the integrity of the graph structure. Based on the matching results, the network establishes two types of connecting edges: “attribution edges” connecting spot nodes with their matched subaperture nodes, and “adjacency edges” connecting spot nodes corresponding to spatially adjacent subapertures. This architectural design intrinsically embeds both the spot-subaperture matching topology and the local spatial correlations of the wavefront into the graph structure, naturally aligning with the physical sampling mechanism of the SHWS. As shown in Fig. 2b, the network processes graph data through stacked multi-head attention layers. Within each layer, nodes perform feature self-attention aggregation from their connected neighboring nodes. The attention mechanism allows each node to dynamically learn the importance weights of different neighbor nodes. Intuitively, neighbors exhibiting similar morphological distortion patterns receive higher attention weights, enabling the model to adaptively aggregate information from relevant subapertures and extract high-precision wavefront phase features from the severely distorted spot group. The node features aggregated through multi-head attention are globally pooled and passed through fully connected layers to directly regress the Zernike coefficients. This method completely avoids the limitations of centroid calculation and ultimately achieves high-precision reconstruction under a large dynamic range.

### Breaking the physical dynamic range limit

**Fig. 4:**
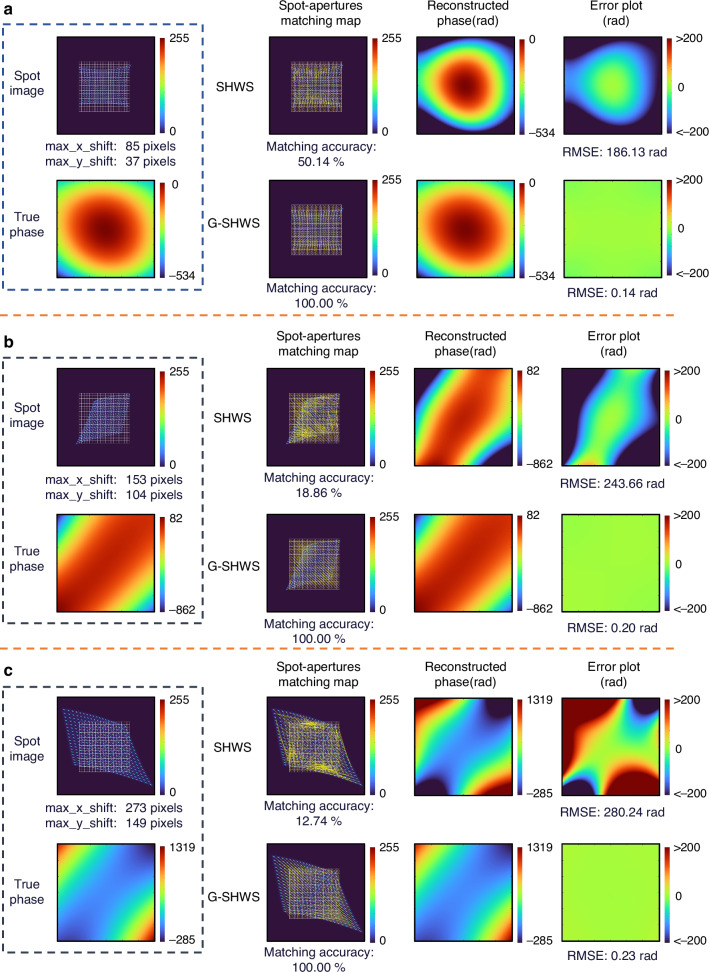
Performance comparison of composite large dynamic range wavefront reconstruction. **a** Maximum displacement 85 pixels (5.6× conventional limit). **b** Maximum displacement 153 pixels (10.2× conventional limit). **c** Maximum displacement 273 pixels (18.2× conventional limit). From left to right: spot image, matching map, reconstructed phase, error plot

To quantify the performance gain, we conduct high-fidelity simulations based on a Shack-Hartmann system with a $19\times 19$ microlens array (pitch size $p=0.15$ mm, focal length $f=5.0{mm}$) and a detector with 5.0 ${\rm{\mu }}m$ pixels. Under these physical constraints, the conventional dynamic range is geometrically limited to a maximum spot displacement of approximately 15 pixels (corresponding to the subaperture boundary). To challenge this boundary, we generate compound large dynamic range wavefronts composed of the first 15 Zernike polynomials, as shown in Fig. 4. These test scenarios feature maximum spot displacements of 85, 153, and 273 pixels—equivalent to 5.6, 10.2, and 18.2 times the conventional limit, respectively. To quantitatively evaluate the reconstruction accuracy, the Root Mean Square Error (RMSE) between the reconstructed wavefront ${W}_{{rec}}$ and the ground truth ${W}_{{true}}$ is calculated as:

1 $${RMSE}=\sqrt{\frac{1}{N}\mathop{\sum }\limits_{i=1}^{N}{\left({W}_{{rec}}\left(i\right)-{W}_{{true}}\left(i\right)\right)}^{2}}$$

where $N$ denotes the total number of valid sampling points within the pupil.

As shown in Fig. 4a, under a displacement of 85 pixels, the spots have significantly deviated from their assigned subapertures and intruded into neighboring regions. This severe crosstalk destroys the regular array previously relied upon by the conventional SHWS, resulting in a matching accuracy of only 50.14% and severe distortion in the reconstructed wavefront, with an RMSE as high as 186.13 rad. In contrast, G-SHWS utilizes the global combinatorial optimization mechanism based on bipartite graphs to achieve a 100% matching success rate without requiring local structural assumptions. Furthermore, by integrating the GAT network with matching topology information, it achieves high-precision reconstruction with an RMSE of only 0.14 rad. As the dynamic range further expands to 153 pixels (Fig. 4b) and 273 pixels (Fig. 4c), the matching rate of the conventional SHWS plummets to below 20%, causing reconstruction to fail completely. Meanwhile, G-SHWS maintains a 100% matching success rate under all test conditions, and the reconstruction RMSE remains below 0.24 rad, demonstrating excellent matching and reconstruction capabilities under a large dynamic range.

**Fig. 5:**
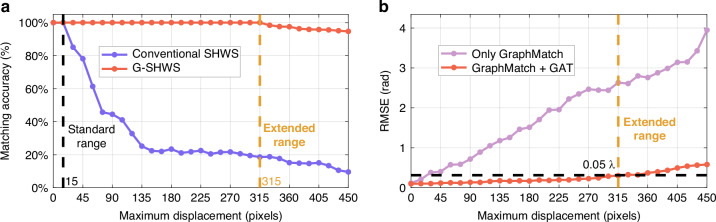
Quantitative analysis of matching accuracy and reconstruction precision versus dynamic range. **a** Matching accuracy curves of conventional SHWS and G-SHWS as a function of maximum spot displacement. The conventional SHWS maintains 100% matching accuracy within 15 pixels, whereas G-SHWS maintains 100% accuracy up to 315 pixels. **b** RMSE curves of wavefront reconstruction by G-SHWS under two configurations (Graph Match only and Graph Match + GAT) as a function of maximum spot displacement. Under the Graph Match + GAT configuration, G-SHWS achieves an RMSE of < 0.05λ within the effectively extended dynamic range

To quantitatively evaluate the performance gain, Fig. 5 analyzes the system performance as the maximum spot displacement varies within the range of 0 to 450 pixels. As shown in Fig. 5a, the conventional SHWS is effective only within the interval where the displacement is less than 15 pixels (i.e., the distance from the subaperture center to its boundary); once this boundary is crossed, its matching accuracy drops precipitously. Conversely, G-SHWS maintains a 100% matching accuracy within a displacement of 315 pixels, successfully extending the measurable dynamic range of the SHWS to 21 times its conventional limit. Additionally, the ablation study in Fig. 5b reveals the necessity of the “Graph Match + GAT” architecture. If only the graph matching strategy is applied while retaining conventional centroiding for slope calculation, the RMSE at 315-pixel displacement remains high at 2.63 rad ($0.42{\rm{\lambda }}$), failing to satisfy the Maréchal criterion ($0.07{\rm{\lambda }}$)^36^. This result exposes a fundamental limitation: severe spot distortion renders the single centroid coordinate an insufficient descriptor for the complex local wavefront curvature. After introducing GAT, the model can explicitly fuse the matching topology with both the spatial distribution and intensity morphology of the spots, mining high-order gradient information from the distorted spot morphology. Consequently, under a dynamic range with a spot displacement of 315 pixels, the RMSE is stably controlled within 0.30 rad ($0.05{\rm{\lambda }}$). This result confirms that G-SHWS not only solves the matching problem under spot crosstalk but also breaks through the physical limitations of the conventional centroid model, effectively balancing dynamic range and reconstruction accuracy. It is worth noting that the factor limiting the further expansion of G-SHWS’s measurable dynamic range beyond 21-fold under composite aberrations is spot splitting. When exceeding this threshold, some focal spots on the detector plane begin to fragment. These split fragments interfere with the characterization of the true spot distribution, leading to incorrect pairing between fitted spots and invalid fragments, causing the sub-aperture attribution of fitted spots to be transferred to invalid split fragments, which causes the matching accuracy to drop below 100%. Therefore, this spot splitting phenomenon defines the upper limit of G-SHWS’s measurable dynamic range.

### Generalization and robustness analysis

Different orders of Zernike aberrations possess unique wavefront gradient field structures, leading to differentiated spot displacement patterns. To evaluate the robustness gain of G-SHWS against various types of aberrations in a decoupled manner, the maximum coefficient amplitude ranges within which the conventional SHWS and G-SHWS can each maintain 100% matching accuracy for single Zernike modes from the 4th to the 15th term are systematically determined.

**Fig. 6:**
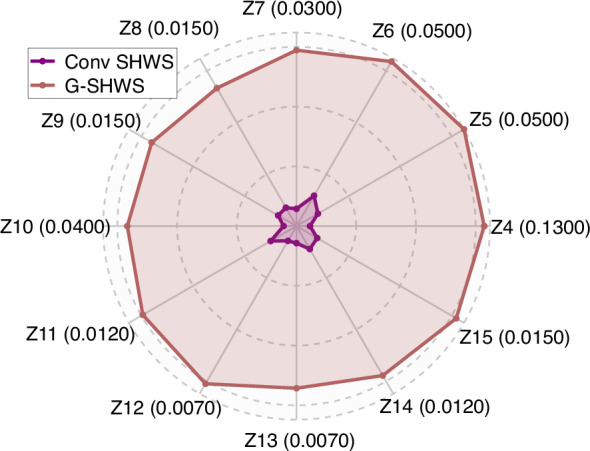
Comparison of dynamic range gain for single Zernike modes. Radar chart comparison of the full measurable span of Zernike coefficients (in mm) for modes Z4–Z15 between Conventional SHWS and G-SHWS

Table 1 details the maximum measurable coefficient amplitude range for each term. Since the spot displacement is linearly correlated with the Zernike coefficients, these coefficient amplitudes directly reflect the upper limit of the system’s effective dynamic range. The radar chart in Fig. 6 clearly reveals the non-uniform gain characteristics of G-SHWS for different aberration patterns: the largest improvement is observed for the 4th term---oblique astigmatism, reaching up to 13.93 times. The reason is that oblique astigmatism drives spots to shift significantly along the diagonal direction, most thoroughly destroying the row-column orthogonality of the microlens array, causing conventional algorithms relying on regular grid priors to fail rapidly. In contrast, the global optimization mechanism of G-SHWS based on graph topology is completely independent of geometric rules, thus demonstrating significant advantages in handling such “strong topological perturbation” aberrations. By comparison, the smallest improvement is for the 6th term---vertical astigmatism, which is 5.42 times. This is because this aberration mainly induces spot movement along the horizontal or vertical axes, partially preserving the row-column orderliness of the array, allowing conventional methods to maintain matching during mild crosstalk. Notably, the measurable dynamic range expansion of G-SHWS for single-type aberrations is smaller than that for composite aberrations. This is because the distortion energy in single-type aberrations is highly concentrated in specific regions, causing the diffraction limit to be exceeded earlier and spot splitting to occur at smaller dynamic ranges, which ultimately leads to algorithm failure.

**Table 1:** Comparison of the measurable dynamic range for single Zernike modes (Z4--Z15)

	Z4	Z5	Z6	Z7	Z8	Z9
	Obl. Astig.	Defocus	Vert. Astig.	Obl. Trefoil	Vert. Coma	Horiz. Coma
SHWS	0.0088	0.0062	0.0088	0.0026	0.0016	0.0016
G-SHWS	0.1226	0.0486	0.0477	0.0265	0.0120	0.0126

	Z10	Z11	Z12	Z13	Z14	Z15
	Horiz. Trefoil	Obl. Quad.	Obl. 2nd Astig.	Spherical	Vert. 2nd Astig.	Horiz. Quad.
SHWS	0.0026	0.0018	0.0006	0.0006	0.0016	0.0018
G-SHWS	0.0340	0.0107	0.0064	0.0057	0.0104	0.0139

The values represent the full measurable span (peak-to-valley) of Zernike coefficients (in mm) for conventional SHWS and G-SHWS to maintain 100% matching accuracy

**Fig. 7:**
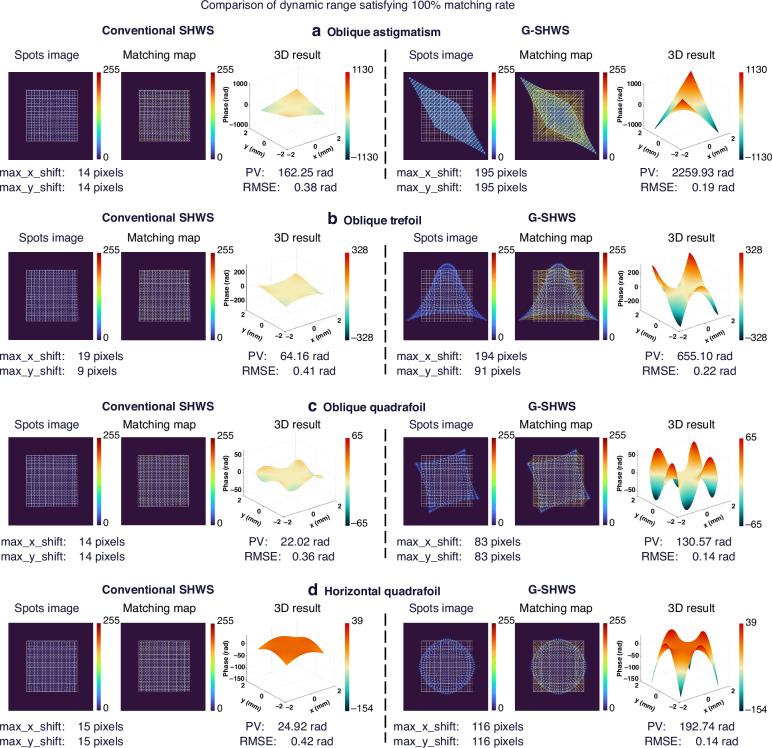
Comparison of dynamic range limits for typical aberrations. Maximum recoverable dynamic range wavefronts by conventional SHWS and G-SHWS under **a** Oblique Astigmatism, **b** Oblique Trefoil, **c** Oblique Quadrafoil, and **d** Horizontal Quadrafoil

Figure 7 intuitively displays the comparison results for four typical high-order aberrations (Oblique Astigmatism, Oblique Trefoil, Oblique Quadrafoil, and Horizontal Quadrafoil) under their limit dynamic ranges. The effective dynamic range of the conventional SHWS is limited to half the subaperture pitch, with maximum measurable spot displacements of 14, 19, 14, and 15 pixels, respectively, corresponding to maximum measurable wavefront PV values of only 162, 64, 22, and 25 rad for the four aberration cases, respectively. In contrast, G-SHWS successfully extends the maximum measurable spot displacements to 195, 194, 83, and 116 pixels, respectively, with corresponding measurable wavefront PV values increasing to 2259, 655, 130, and 192 rad, intuitively reflecting the enhancement in its measurement capability for various complex aberrations. Furthermore, within such a substantially expanded measurement interval, the wavefront reconstruction RMSE of G-SHWS is stably controlled between 0.14 and 0.23 rad. This precision performance is superior to that of the conventional SHWS under a small dynamic range, validating the high-precision characteristics of G-SHWS when dealing with various aberrations.

In real-world complex wavefront measurement scenarios, aberrations often contain spatially stochastic disturbances. Strong atmospheric turbulence serves as a representative instance of such perturbations. It is generally defined as turbulence with an atmospheric refractive index structure constant ${C}_{n}^{2}$ greater than ${10}^{-14}{m}^{-2/3}$ ^37^, inducing severe wavefront distortions and large local slopes. To validate the adaptability of G-SHWS in such complex scenarios and enable quantitative comparison with precise ground truth, we use phase screens simulating strong atmospheric turbulence to conduct comparative reconstruction experiments. These phase screens are generated based on the Kolmogorov turbulence spectrum via a frequency-domain synthesis method^38^. Specifically, the power spectral density is inverted in combination with the Fast Fourier Transform (FFT) to produce wavefronts satisfying the requisite statistical properties. Given that turbulence wavefronts contain rich high spatial frequency components, both the conventional SHWS and G-SHWS are configured to use 45 Zernike polynomials for wavefront fitting, which represents a trade-off between characterization capability and the spatial sampling limit of the microlens array. Higher-order terms would exceed the sensor’s effective spatial bandwidth and introduce fitting instability.

**Fig. 8:**
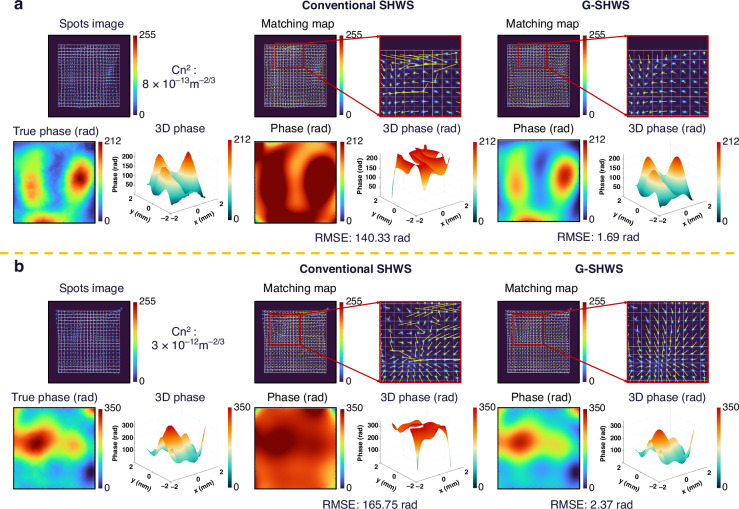
Comparison of matching maps and wavefront reconstruction results under atmospheric turbulence. **a** Turbulence scenario with ${C}_{n}^{2}=8\times 1{0}^{-13}\,{\mathrm{m}}^{-2/3}$. **b** Turbulence scenario with ${C}_{n}^{2}=3\times 1{0}^{-12}\,{\mathrm{m}}^{-2/3}$

Figure 8 shows two strong turbulence scenarios with ${C}_{n}^{2}$ of $8\times {10}^{-13}{m}^{-2/3}$ and $3\times {10}^{-12}{m}^{-2/3}$, respectively. As illustrated in Fig. 8a, b, large local gradients cause significant spot aliasing and distortion. Consequently, the conventional SHWS exhibits chaotic matching, yielding RMSEs of 140.33 rad and 165.75 rad, respectively, which indicates a complete failure in measurement capability. In contrast, G-SHWS successfully reconstructs the correct matching relationships despite severe aliasing and effectively extracts aberration information from the distorted spot intensity morphology. It achieves RMSEs of only 1.69 rad and 2.37 rad. These values represent a reduction of over 98% compared to the conventional SHWS, demonstrating the advantages of G-SHWS in strong turbulence scenarios. It is worth noting that the RMSE of G-SHWS in this test is significantly higher than that in the previous two simulation experiments. This arises because a portion of the extremely high-frequency aberrations within the strong turbulence exceeds both the limited spatial sampling resolution of the microlens array and the fitting capacity of the 45 Zernike polynomials, thereby introducing an inherent accuracy limit.

**Fig. 9:**
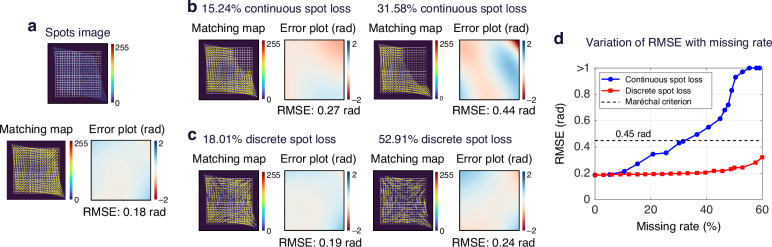
Analysis of matching results and reconstruction errors of G-SHWS under different spot loss conditions. **a** Matching results and reconstruction error under the original spot image. **b** Results under continuous spot loss conditions with missing rates of 15.24% and 31.58%. **c** Results under discrete spot loss conditions with missing rates of 18.01% and 52.91%. **d** Variation of RMSE with missing rate under continuous and discrete spot loss conditions

Spot loss is a common phenomenon in SHWS measurements caused by field-of-view occlusion. To verify the robustness of G-SHWS under spot loss conditions, we select a high dynamic range spot image for reconstruction under both continuous and discrete loss scenarios. As shown in Fig. 9a, under non-loss conditions, the reconstruction RMSE of G-SHWS for this wavefront is 0.18 rad. As depicted in Fig. 9b, we apply continuous occlusion to the upper-right direction of the spot array, creating spot loss rates of 15.24% and 31.58%, respectively. G-SHWS maintains precise spot-to-subaperture matching under both loss rates, benefiting from the introduction of high-cost virtual nodes into the cost matrix during the matching phase, which acts as a penalty filter to automatically identify and discard unmatched nodes caused by spot loss. Furthermore, leveraging the dynamic node filling mechanism during the reconstruction phase, the RMSE remains at 0.27 rad and 0.44 rad, respectively, still satisfying the Maréchal criterion (0.45 rad). The increase in reconstruction error stems from subtle differences between the features of the fitted spot nodes filled in the missing regions and those of actual spots. As shown in Fig. 9c, when facing discrete loss, G-SHWS demonstrates superior robustness; even at a loss rate of 52.91%, the RMSE increases only to 0.24 rad (0.038${\rm{\lambda }}$). This is because, under discrete spot loss, the remaining actual spots preserve the global spatial distribution pattern, providing strong constraints for the GAT’s node feature aggregation. During the GAT’s feature aggregation process, the features of the fitted spots filled in missing regions can be more effectively optimized by the features of surrounding actual spot nodes, thereby facilitating the GAT to regress higher-precision results. The line chart in Fig. 9d intuitively reflects this: the effective robustness of G-SHWS against continuous loss is maintained up to approximately 35% loss rate, while under discrete loss, it extends even to a 60% loss rate. This robustness against spot loss significantly enhances the measurement capability of SHWS under non-continuous sampling conditions.

### Experimental validation

To verify the performance of G-SHWS in real physical scenarios, we built an SHWS system. The system includes a microlens array (MLA, Thorlabs MLA1507AR, subaperture pitch $p=150$ ${{\rm{\mu }}m}$) and a CMOS sensor (Sony IMX249, pixel size 5.86 ${{\rm{\mu }}m}$). Using the spherical wavefront reference calibration method^39^, the distance between the MLA and the sensor plane was precisely calibrated to be 5.28 mm. Under this configuration, the theoretical dynamic range limit of the conventional SHWS corresponds to a maximum spot displacement of 12.8 pixels, which translates to a maximum measurable wavefront slope threshold of 0.0142 rad.

**Fig. 10:**
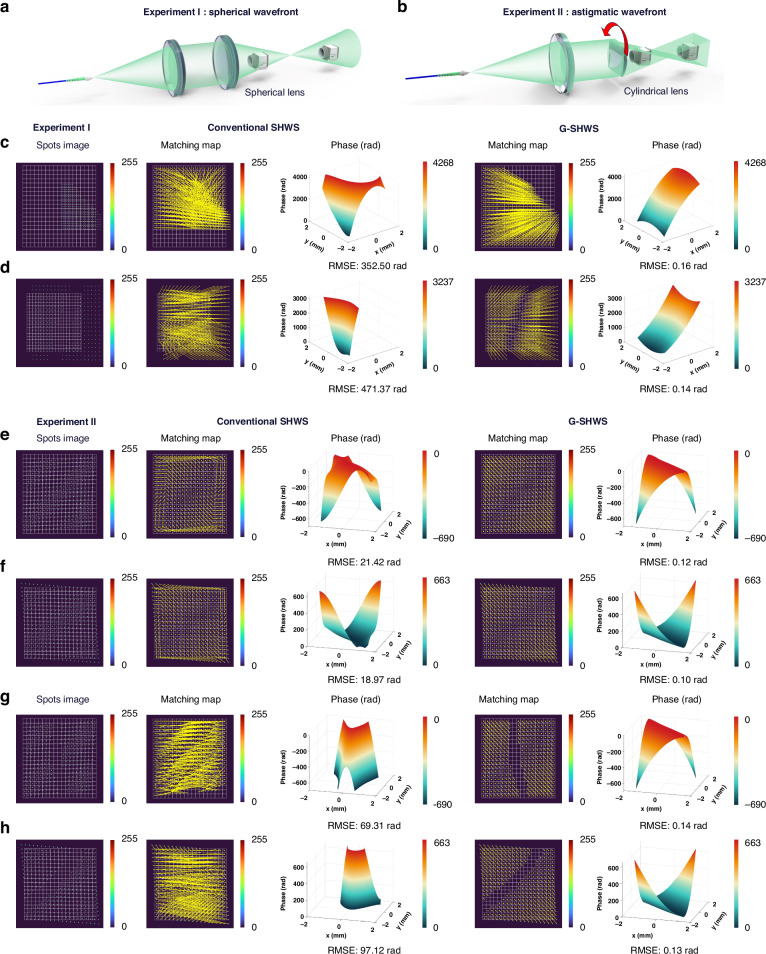
Experimental validation of large dynamic range wavefront measurement. **a** Optical setup for measuring high-slope spherical wavefronts. **b** Setup for astigmatic wavefronts using a rotatable cylindrical lens. **c**, **d** Results for obstructed spherical wavefronts under converging and diverging conditions. **e**, **f** Results for anisotropic astigmatism (50° rotation) under converging and diverging conditions. **g**, **h** Results for obstructed astigmatism

First, we design a set of high-curvature spherical wave experiments aimed at quantitatively evaluating the dynamic range expansion capability of G-SHWS. As shown in Fig. 10a, a beam emitted by a 532 nm single-mode fiber laser is collimated by a lens and then focused by a lens with a focal length of 150 mm. We place the sensor at defocus positions before and after the focal point (converging and diverging regions) and perform lateral off-axis movement, thereby introducing spherical wavefronts with extremely large local slopes and asymmetric distributions. Additionally, we introduce opaque filamentary obstacles in the optical path to artificially block some subapertures, simulating non-continuous sampling scenarios under interference conditions.

As shown in Fig. 10c, d, under the dual effects of deep defocus and off-axis alignment, the spot array undergoes severe inward and outward crosstalk as well as overall displacement. Furthermore, spot loss rates of 24.9% and 17.7% are generated due to subaperture occlusion, respectively. The conventional SHWS fails to match when the spot displacement exceeds the subaperture boundary and spot loss occurs. In both converging and diverging tests, the reconstruction RMSE calculated against the best-fit spherical surface reaches as high as 352.50 rad and 471.37 rad, respectively, resulting in a complete loss of wavefront reconstruction capability. In contrast, relying on the global combinatorial optimization mechanism based on the bipartite graph minimum cost flow, G-SHWS not only achieves an unambiguous global optimal matching under strong spot crosstalk conditions but also automatically identifies the subapertures corresponding to missing spots by introducing high-cost unmatched terms. On this basis, the GAT in the reconstruction stage further utilizes the converged estimated wavefront to generate proxy spot features, performing physically consistent filling for missing nodes. This maintains the integrity of the graph structure, allowing the reconstructed wavefront to accurately restore the spherical characteristics, yielding RMSEs relative to the best-fit spherical surface of only 0.16 rad and 0.14 rad in converging and diverging cases, respectively.

Calculations indicate that for the two spherical wavefronts successfully measured by G-SHWS above, the maximum spot displacements generated in the full spot array reach 259 pixels and 180 pixels, respectively. The corresponding maximum local wavefront slopes are 0.2874 rad and 0.1998 rad, reaching 20.24 times and 14.07 times the measurable wavefront slope limit of the conventional SHWS. Under these extreme parameters, the PV values of the two spherical waves reach as high as $679.6{\rm{\lambda }}$ and $515.4{\rm{\lambda }}$, respectively, validating that G-SHWS significantly enhances the measurable range and robustness of SHWS in real-world scenarios.

To further test the adaptability of G-SHWS to complex topological structures, as shown in Fig. 10b, we replace the focusing lens with a cylindrical lens of focal length $f=60$ mm and rotate its principal axis counterclockwise by ${50}^{\circ }$. This setup introduces significant oblique astigmatism. Unlike the radial symmetry of spherical waves, this aberration drives spots to undergo drastic unidirectional stretching and systematic tilting along non-orthogonal directions. As shown in the spot images in Fig. 10e, f, in the converging region, spots cluster densely inward along the oblique direction; in the diverging region, they spread outward along the same direction. This anisotropic non-orthogonal gradient distribution destroys the “row-column orthogonal grid” distribution of the conventional spot array. Since the phase modulated by the cylindrical lens strictly belongs to the quadratic function space under the paraxial approximation, its theoretical wavefront can be fully described by low-order aberrations such as defocus and astigmatism. Therefore, we perform a best-fit quadric surface for the wavefronts reconstructed by both the conventional SHWS and G-SHWS, and calculate the RMSE between the reconstructed wavefront and the fitted surface as a metric to quantitatively evaluate reconstruction quality.

As shown in the comparison results in Fig. 10e, f, the conventional SHWS exhibits numerous mismatching errors in the edge regions where spots are severely compressed and stretched, leading to severe local distortion in the reconstructed wavefront. In both converging and diverging states, its reconstruction RMSE reaches as high as 21.42 rad and 18.97 rad, respectively. In contrast, G-SHWS achieves precise matching while perfectly decoupling the non-orthogonal wavefront information. It significantly reduces the RMSE to 0.12 rad and 0.10 rad, respectively, demonstrating excellent direction-independent robustness.

Furthermore, similar to the operation in the spherical wavefront experiment, we introduce obstacles in front of the sensor, as shown in Fig. 10g, h, causing spot loss rates of 13.0% and 14.4%, respectively. At this point, the conventional SHWS suffers from global matching collapse triggered by local aperture failure, with RMSE surging to 69.31 rad and 97.12 rad, completely losing the quadric surface characteristics. Meanwhile, G-SHWS still effectively identifies missing nodes during matching and fills them in the reconstruction stage, maintaining the consistency of the global topology. The final RMSE only increases marginally to 0.14 rad and 0.13 rad.

The measurement results for large-slope spherical and cylindrical wavefronts indicate that G-SHWS not only solves the range issue of dynamic range but also fundamentally overcomes the inherent limitation of SHWS being only capable of measuring wavefronts with approximately regular gradient distributions, possessing the general capability to handle complex aberrations and non-continuous sampling.

## Discussion

This paper proposes G-SHWS, a graph-theoretic computational model designed to overcome the dynamic range limitations of Shack-Hartmann wavefront sensors. G-SHWS comprises two core components. First, it employs a bipartite graph global combinatorial optimization strategy to reconstruct the global spot-subaperture matching relationship. This mechanism effectively resolves matching ambiguities caused by crosstalk, ensuring matching robustness under severe spot aliasing and spot loss. Second, a GAT embedded with matching topology priors is constructed. The GAT explicitly encodes the aforementioned matching relationships via the graph structure and effectively utilizes spot spatial features and intensity distribution morphology through a graph attention aggregation mechanism. This facilitates high-precision reconstruction of large dynamic range wavefronts, transcending the representation limitations of conventional single-vector centroiding under large aberrations.

Both simulation and experimental results demonstrate that G-SHWS extends the measurable range of conventional SHWS by 21 times, achieving high-precision wavefront reconstruction under conditions of severe spot crosstalk and distortion. And it maintains exceptional robustness in the presence of extensive spot loss. Without requiring any hardware modifications, this approach significantly enhances the capability of SHWS to measure various complex aberrations and improves its adaptability to non-ideal operating conditions. This graph-theoretic modeling and solving strategy for SHWS offers a computational solution for demanding applications such as astronomical adaptive optics, aerodynamic optical aberration detection, and high-order ocular aberration diagnosis.

Future work on this study could further enhance the performance of G-SHWS from multiple perspectives. Regarding the dynamic range, the primary factor limiting further expansion of G-SHWS is the spot splitting phenomenon occurring under larger dynamic ranges, as previously mentioned. In the future, specialized segmentation algorithms could be designed to accurately extract the main body of the spots, thereby preventing split fragments from affecting the matching iteration. Additionally, dedicated feature extraction operators could be developed to effectively encode the multi-peak structure and discrete intensity characteristics of split spots, quantizing them as node features within the GAT for network regression. Through these two improvements, the dynamic range of G-SHWS is expected to be further extended.

In terms of computational efficiency, the current system leverages the temporal continuity of wavefront variations, employing an adjacent-frame initialization strategy (see Materials and methods: Implementation details and computational efficiency) to significantly reduce the iterations required for matching. Since the current computational time is primarily consumed by the Minimum Weight Perfect Matching (MWPM) on bipartite graphs within each iteration of the matching stage, future research could explore MWPM solvers with lower algorithmic complexity. This would significantly reduce the computational overhead per iteration and ultimately enhance G-SHWS’s adaptability to measurement scenarios with extremely stringent real-time requirements.

## Materials and methods

### Global matching strategy

To recover the global matching topology under conditions of severe spot crosstalk and a lack of local priors, we treat the actual spot set $T=\{{t}_{1},\ldots ,{t}_{m}\}$ and the fitted spot set $F=\{{f}_{1},\ldots ,{f}_{n}\}$ as two independent node sets to construct a weighted bipartite graph model. The estimated wavefront $W\left(x,y\right)$ is parameterized using the first $K=15$ Zernike polynomials, which is sufficient to describe the deformation characteristics of most wavefronts:

2 $$W\left(x,y\right)=\mathop{\sum }\limits_{k=1}^{K}{C}_{k}{Z}_{k}\left(x,y\right)$$

where ${Z}_{k}$ and ${C}_{k}$ denote the $k$-th Zernike polynomial and its coefficient. The numerical representation $\left({x}_{f}^{\left(i\right)},{y}_{f}^{\left(i\right)}\right)$ of the fitted spot distribution is calculated using the following SHWS paraxial imaging model:

3 $$\left\{\begin{array}{c}{x}_{f}^{\left(i\right)}={x}_{r}^{\left(i\right)}+f\sum {C}_{k}\frac{\partial {Z}_{k}}{\partial x}\\ {y}_{f}^{\left(i\right)}={y}_{r}^{\left(i\right)}+f\sum {C}_{k}\frac{\partial {Z}_{k}}{\partial y}\end{array}\right.$$

where $\left({x}_{r}^{\left(i\right)},{y}_{r}^{\left(i\right)}\right)$ denotes the reference centroid coordinates of the $i$-th microlens, and $f$ is the focal length. The numerical representation of the actual spot distribution is obtained by segmenting the actual spot image using the maximum inter-class variance method (Otsu’s method)^40^ and extracting the centroid $\left({x}_{a}^{\left(i\right)},{y}_{a}^{\left(i\right)}\right)$ of each connected region. The edge weights in the bipartite graph are defined by the Euclidean distances between all node pairs across the two node sets, generating an $m\times n$ cost matrix $C$:

4 $$\left.C=\left[\begin{array}{cccc}{c}_{1,1} & {c}_{1,2} & \cdots & {c}_{1,n}\\ \vdots & \vdots & \ddots & \vdots \\ {c}_{m,1} & {c}_{m,2} & \cdots & {c}_{m,n}\end{array}\right.\right]$$

where the element ${c}_{{i,j}}={\left|\left|{t}_{i}-{f}_{j}\right|\right|}_{2}$ represents the distance between the $i$ th actual spot node and the ${j}$ -th fitted spot node. Subsequently, the Jonker-Volgenant algorithm is employed to rapidly find the fitted-actual spot pairing with the minimum global pairing cost within the cost matrix under the current fitted Zernike coefficients by constructing an equivalent dual problem and optimizing the potential function (see Supplementary Note 1 for details). The resulting minimum total distance is returned as the pairing cost:

5 $${\mathscr{L}}=\mathop{\min }\limits_{\pi \in \Pi }\,\mathop{\sum }\limits_{i=1}^{m}\,{c}_{i,\pi \left(i\right)}$$

where $\Pi$ is the set of injective mappings from $\{1,\ldots ,m\}$ to $\{1,\ldots ,n\}$. It is worth noting that when spot loss causes an inconsistency in the cardinality of the actual and fitted node sets ($m\ne n$), we construct an augmented cost matrix ${C}_{\mathrm{aug}}$ of size $\left(m+n\right)\times \left(m+n\right)$ as shown in the following equation:

6 $${C}_{{\rm{aug}}}=\left[\begin{array}{cc}C & 2\lambda \cdot {I}_{m}\\ 2\lambda \cdot {I}_{n} & {C}^{T}\end{array}\right]$$

where ${C}^{{\rm{\top }}}$ denotes the transpose of $C$. The matrices ${I}_{m}{and}{I}_{n}$ are diagonal matrices of size $m\times m$ and $n\times n$ respectively, with ones on the diagonal and infinity ($\infty$) elsewhere. The parameter ${\rm{\lambda }}$ (${\rm{\lambda }}={10}^{6}$) serves as the penalty threshold for unmatched spots. This construction enables the Jonker-Volgenant algorithm to automatically assign excess fitted spots to virtual nodes, selecting a subset from the fitted spots that optimally pairs with the $m$ actual spots. This effectively eliminates redundant fitted spots in the output matching pairs, thereby achieving global optimal pairing even under spot loss conditions.

Subsequently, the Atom Search Optimization (ASO) algorithm (see Supplementary Note 2 for details) is adopted to iteratively update the estimated wavefront parameter set in the Zernike coefficient space $\{{C}_{k}{\}}_{k=1}^{K}$, thereby causing changes in the fitted spot distribution generated by this set. ASO performs updates in the direction of reducing the pairing cost between fitted and actual spots, driving the fitted spot distribution to approximate the actual distribution. When the pairing cost converges below a predefined threshold, the fitted spot distribution achieves geometric alignment with the actual spot distribution. At this point, the known subaperture attributions of the fitted spots are mapped to their paired actual spots, thereby enabling precise spot-subaperture matching. To avoid spending time searching in meaningless regions, the search ranges for the 1st to 6th terms are set to $\left[-\mathrm{0.3,0.3}\right]$ mm, the 7th to 10th terms to $\left[-\mathrm{0.1,0.1}\right]$ mm, and the 11th to 15th terms to $\left[-\mathrm{0.05,0.05}\right]$ mm. At the beginning of iteration, all Zernike coefficients are initialized to 0 to provide a neutral starting point.

### GAT-based reconstruction network

To fully utilize the high-order aberration information inherent in spot intensity morphology and explicitly fuse the global matching results, we propose a Graph Attention Network (GAT) incorporating the SHWS matching topology.

The network abstracts actual spots and their corresponding subapertures as graph nodes. The feature vector of each actual spot node is a 14-dimensional embedding comprising: (1) 4-dimensional spatial extreme features, representing the boundary coordinates of the connected region $\{{x}_{\min }/f,{x}_{\max }/f,{y}_{\min }/f,{y}_{\max }/f\}$; and (2) 10-dimensional morphological descriptors, encompassing normalized second-order central moments $\{{{\rm{\mu }}}_{20},{{\rm{\mu }}}_{02},{{\rm{\mu }}}_{11}\}$ and Hu invariant moments $\{{h}_{1},\ldots ,{h}_{7}\}$, which collectively characterize the asymmetric intensity distribution. Correspondingly, each subaperture node is encoded with a 2-dimensional angular domain feature $\left({x}_{r}/f,{y}_{r}/f\right)$, representing its reference centroid position. To ensure graph completeness, for missing spots identified during matching, we generate proxy spot images using the converged estimated wavefront. Their features are extracted via the identical process to maintain structural integrity. Subsequently, the graph topology is constructed based on the global matching results. Two specific types of edges are established to implement physical constraints: (1) attribution edges connecting each actual spot node to its corresponding subaperture node, designed to explicitly encode the recovered matching relationship; and (2) spatial adjacency edges connecting spot nodes corresponding to spatially adjacent subapertures, designed to preserve physical spatial neighborhood relationships to introduce the local spatial correlations of the wavefront gradient field. This architecture naturally aligns with the physical sampling mechanism of the SHWS, allowing the network to effectively fuse matching topology, spot spatial distribution, and morphological information.

As shown in Fig. 2b, the first layer of the network concatenates the features of each actual spot node with those of its corresponding subaperture node to form a 16-dimensional initial aggregated node. This is mapped to a 16-dimensional latent space via a learnable linear transformation, serving as the input for the subsequent graph attention layers. Subsequently, four multi-head graph attention layers are stacked. In the 2nd to 4th layers, a multi-head attention mechanism is employed to efficiently compute $K=4$ independent attention heads in parallel. Each head independently calculates attention weights and computes a weighted sum of neighbor features to obtain the updated node features under that head:

7 $${h}_{v}^{{\prime} \left(k\right)}={\rm{\sigma }}\left(\mathop{\sum }\limits_{{\rm{u}}\in {\mathscr{N}}\left({\rm{v}}\right)}{{\rm{\alpha }}}_{{vu}}^{\left(k\right)}{W}^{\left(k\right)}{h}_{u}\right),\,k=1,\ldots ,K$$

where ${h}_{u}$ is the current feature of the neighbor node, ${\mathscr{N}}\left(v\right)$ represents the set of neighbor nodes of node $v$, ${W}^{\left(k\right)}$ is the learnable weight matrix for the $k$-th head, ${\rm{\sigma }}$ is the ELU activation function, and ${{\rm{\alpha }}}_{{vu}}^{\left(k\right)}$ is the attention coefficient between node $v$ and $u$ in the $k$-th head, calculated as:

8 $${{\rm{\alpha }}}_{{vu}}^{\left(k\right)}=\frac{\exp \left(\mathrm{LR}\left({a}^{\left(k\right)T}\left[{W}^{\left(k\right)}{h}_{v}|{|W}^{\left(k\right)}{h}_{u}\right]\right)\right)}{{\sum }_{n\in {\mathscr{N}}\left(v\right)}\exp \left(\mathrm{LR}\left({a}^{\left(k\right)T}\left[{W}^{\left(k\right)}{h}_{v}{||}{W}^{\left(k\right)}{h}_{n}\right]\right)\right)}$$

where $\mathrm{LR}\left(\cdot \right)$ denotes the LeakyReLU, ${||}$ represents vector concatenation and ${a}^{\left(k\right)}$ is a learnable parameter vector. Since spot morphology exhibits strong correlations with local wavefront distortion, this attention mechanism enables the model to dynamically learn the contribution weights of different neighbor nodes based on feature similarities. Intuitively, neighbors exhibiting similar morphological distortion patterns receive higher attention weights, allowing the model to aggregate information from highly correlated subapertures and thereby extract wavefront phase features more precisely from the severely distorted spot group. Subsequently, the outputs of all $K$ heads are concatenated to form a diversified feature representation, yielding the final output feature of the layer:

9 $${h}_{\nu }^{{\prime} }={{\rm{||}}}_{k=1}^{K}{h}_{\nu }^{{\prime} }\left(k\right)$$

In the 5th layer (output layer), the outputs of $K=4$ attention heads are also calculated, but they are fused via average pooling to achieve a smoother and more stable global representation:

10 $${h}_{v}^{{\prime} }={\rm{\sigma }}\left({W}_{{out}}\left(\frac{1}{K}\mathop{\sum }\limits_{k=1}^{K}\mathop{\sum }\limits_{u\in {\mathscr{N}}\left(v\right)}{{\rm{\alpha }}}_{{vu}}^{\left(k\right)}{W}^{\left(k\right)}{h}_{u}\right)\right)$$

where ${W}_{{out}}\in {R}^{64\times 16}$ represents a learnable linear transformation matrix used to map features from the 16-dimensional space to a 64-dimensional space. Finally, global average pooling is performed on the 64-dimensional features of all spot nodes to obtain a 64-dimensional graph-level representation. This representation is then input into a Multi-Layer Perceptron (MLP) containing two hidden layers with dimensions of 32 and 16, respectively, and an output layer dimension of 15. The hidden layers use ReLU activation functions, and the output layer is a linear mapping, finally outputting the 15-dimensional Zernike coefficients to complete the wavefront reconstruction. To more intuitively demonstrate the complete workflow of G-SHWS, we provide a demo video in the Supplementary Materials.

### Implementation details and computational efficiency

To construct a high-fidelity dataset, this paper establishes a physical simulation model of the SHWS based on scalar diffraction theory. The incident wavefront is generated by a random combination of the first 15 Zernike polynomials. The microlens array (MLA) is modeled using the thin lens approximation for phase modulation. The optical field propagation employs the band-limited angular spectrum method^41^ to simulate the free-space diffraction process from the MLA to the CMOS plane, and finally, the squared modulus of the optical field is calculated to obtain the spot intensity image. The specific system parameters are detailed in Table 2.

**Table 2:** Specific parameters of the SHWS used in generating the simulation data

Parameters	Value
Wavelength (nm)	532
Pitch size of micro lens (mm)	0.15
MLA size	19 × 19
Focal length (mm)	5.00–6.00
Camera pixel size (*μ*m)	5.00

The dataset contains 20,000 training samples and 5000 validation samples. For each sample, spot-subaperture matching is completed using the matching mapping method based on bipartite graph global combinatorial optimization, and a graph structure containing an adjacency matrix (encoding matching and spatial neighborhood relationships) and a node feature matrix (containing 14-dimensional spot features and 2-dimensional subaperture position features) is constructed as the network input. To enhance the model’s robustness to spot loss, we randomly apply zero-masking to 10–30% of the area in 50% of the samples. The regression target of the network is the 15-dimensional Zernike coefficients. Considering that the amplitude of low-order aberration coefficients is usually significantly larger than that of high-order aberrations, we adopt a Weighted Mean Squared Error (WMSE) loss function to avoid insufficient fitting of high-order details by the model:

11 $${L}_{{WMSE}}=\frac{1}{N}\mathop{\sum }\limits_{i=1}^{N}\mathop{\sum }\limits_{j=1}^{15}{w}_{j}{\left({z}_{{ij}}-{\hat{z}}_{{ij}}\right)}^{2},\,{w}_{j}=\frac{1}{{{\rm{\sigma }}}_{j}^{2}}$$

where $N$ is the number of samples in a batch, ${z}_{{ij}}$ and ${\hat{z}}_{{ij}}$ represent the $j$ th ground truth and predicted Zernike coefficients of the $i$ -th sample, respectively, and ${w}_{j}$ is the weight coefficient for the $j$ th term. ${{\rm{\sigma }}}_{j}^{2}$ is the variance of the $j$ th coefficient in the training set. Since high-order terms have a smaller amplitude range, their corresponding variance is significantly lower than that of low-order terms; therefore, this weighting strategy assigns higher loss weights to high-order aberrations with smaller variances, thereby enhancing the model’s sensitivity to high-order aberration reconstruction. The model is based on the PyTorch framework, uses the Adam optimizer (initial learning rate 5e−4), is trained on an NVIDIA A100 GPU, and tested on an RTX 4070Ti.

Computational efficiency is a critical factor for practical applications. The optimization process for spot-subaperture matching requires approximately 50 iterations ($\sim$247 ms) for a completely unknown initial large dynamic range wavefront. However, given that wavefront variations in practical applications typically possess temporal continuity, utilizing the reconstructed coefficients from the previous frame as initialization reduces the iterations to fewer than 10 ($< 49\mathrm{ms}$). Furthermore, our GAT network demonstrates high inference speed, stabilizing at approximately 7 ms. Consequently, the total processing time per frame is reduced to within 56 ms, effectively meeting the real-time reconstruction requirements of most dynamic wavefront sensing scenarios.

## Supplementary information

SUPPLEMENTAL MATERIAL

Supplementary Video

## Data Availability

The relevant datasets of this study are available from the corresponding author upon reasonable request.
