# Supplementary material for: Large dynamic range Shack-Hartmann wavefront sensing based on a graph-theoretic computational model
Source: Light Sci Appl. 2026 Apr 15;15:199. doi: 10.1038/s41377-026-02273-x (PMC13079868; doi:10.1038/s41377-026-02273-x)
Supplement: Supplementary file 1 — SUPPLEMENTAL MATERIAL [file 41377_2026_2273_MOESM1_ESM.docx]

**Supplementary information for large dynamic range Shack-Hartmann wavefront sensing based on a graph-theoretic computational model**

### Lintong Du^1 2∗^ , Rui Xu^3 4 ∗^ , Shuxin Liu^1 2 5^ , Rongjun Shao^1 2 5^ , Lin Li^1 2^ , Yuhang Zhang^1 2^, Ziqiang Li^1 2^ , Yuan Qu^1 2 5^ , Dapeng Tian^3 4 †^ , and Jiamiao Yang^1 2 5 †^

### School of Automation and Intelligent Sensing, Shanghai Jiao Tong University, Shanghai, 200240, China

### State Key Laboratory of Submarine Geoscience, Shanghai Jiao Tong University, Shanghai, 200240, China

### State Key Laboratory of Dynamic Optical Imaging and Measurement, Changchun Institute of Optics, Fine Mechanics and Physics, Chinese Academy of Sciences, Changchun 130033, China

### University of Chinese Academy of Sciences, Beijing 100049, China

### Institute of Medical Robotics, Shanghai Jiao Tong University, Shanghai, 200240, China

### ∗These authors contributed equally to this work.

### †Correspondence to: Jiamiao Yang: jiamiaoyang@sjtu.edu.cn, Dapeng Tian: d.tian@ciomp.ac.cn

[Supplementary Note 1: Detailed Jonker-Volgenant algorithm 2](#_Toc223890761)

[Supplementary Note 2: Detailed Atom Search Optimization (ASO) algorithm 3](#_Toc223890762)

[Reference 5](#_Toc223890763)

# Supplementary Note 1: Detailed Jonker-Volgenant algorithm

## S1. Mathematical Formulation: Linear Assignment with Outlier Rejection

As stated in the " Materials and methods: Global matching strategy" section of the main text, to quantify the geometric similarity between the fitted and actual spot distributions, it is necessary to solve for the global optimal pairing between the two sets of spots such that the sum of Euclidean distances of all spot pairs is minimized. This minimum sum of distances directly characterizes the degree of alignment in spatial distribution between the two sets of spots. To this end, we formulate the optimal pairing problem between the fitted spot set and the actual spot set as a Linear Assignment Problem (LAP). To robustly handle complex scenarios involving optical occlusion, or detection failures—where the cardinality of the actual spot set ($m$) differs from that of the fitted spot set ($n$)—we employ a generalized formulation capable of outlier rejection.

To explicitly model the cost of leaving a spot unmatched, we construct a symmetric augmented cost matrix $C_{aug}$ of size $\left( m+n \right)\times\left( m+n \right)$:

$$\begin{aligned} C_{aug}=\left[ \begin{matrix} C & 2\lambda\cdot I_{m} \\ 2\lambda\cdot I_{n} & C^{T} \end{matrix} \right]\#\left( 1 \right) \end{aligned}$$

Here, $C$ denotes the $m \times n$ Euclidean distance matrix between the centroids of actual and fitted spots. The submatrices $I_{m}$ and $I_{n}$ act as auxiliary blocks with diagonal elements set to 1 and off-diagonal elements set to infinity ($\infty$). The parameter $\lambda$ represents the penalty threshold for an unmatched assignment (set to ${1\times10}^{6}$ in our implementation). This symmetric structure guarantees that solving for a perfect matching in $C_{aug}$ is mathematically equivalent to finding the optimal partial matching in $C$ that minimizes the sum of matching distances and penalty terms for unassigned nodes.

## S2. Solver Algorithm: The Jonker-Volgenant Approach

To solve the perfect matching problem on $C_{aug}$, we utilize the Jonker-Volgenant algorithm^1^, a computationally efficient realization of the primal-dual optimization framework (classically referred to as the Hungarian method^2^). The algorithm guarantees global optimality by iteratively updating dual variables (also known as potential functions), denoted as $u_{i}$ for row nodes and $v_{j}$ for column nodes. The optimization process proceeds as follows:

1. Dual Initialization (Column Reduction)

The algorithm begins by initializing the potentials to satisfy the feasibility constraint $u_{i}+v_{j}\leq\left( C_{aug} \right)_{ij}$. To accelerate convergence, a column reduction heuristic is applied:

$$\begin{aligned} u_{i}=0, v_{j}={\min_{i} \left( C_{aug} \right)}_{ij}\#\left( 2 \right) \end{aligned}$$

2. Augmenting Path Search

The algorithm constructs an Equality Subgraph containing only "tight" edges where the reduced cost is zero: $\bar{C_{ij}}=\left( C_{aug} \right)_{ij}-u_{i}-v_{j}=0$. It attempts to find an augmenting path—a path of alternating matched and unmatched edges—within this subgraph to increase the cardinality of the matching. This search is effectively implemented using a shortest-path scan on the residual graph.

3. Potential Function Optimization (Dual Update)

If no augmenting path is found within the current Equality Subgraph, the algorithm performs a dual update to expand the search space. It identifies the set of visited rows $S$ and visited columns $T$ during the failed search and computes the minimum slack $\Delta$:

$$\begin{aligned} \Delta=\min\{\left( C_{aug} \right)_{ij}-u_{i}-v_{j}\mid i\in S,j\notin T\}\#\left( 3 \right) \end{aligned}$$

Subsequently, the potentials are updated to strictly improve the dual objective function while maintaining feasibility:

$\left\{\begin{matrix} u_{i}\leftarrow u_{i}+\Delta& \text{if }i\in S \\ v_{j}\leftarrow v_{j}-\Delta& \text{if }j\in T \end{matrix}\right.$ (4)

This step specifically corresponds to the "optimizing potential function" mechanism mentioned in the main text. By adjusting the potentials, at least one new edge is introduced into the Equality Subgraph, allowing the search for an augmenting path to proceed.

4. Termination

The augmentation and update steps are repeated until the matching cardinality equals the dimension of the augmented matrix (i.e., $m+n$ unique pairings are formed). In this state, every node—whether actual, fitted, or auxiliary—is assigned exactly one partner. Finally, the algorithm parses this global solution: assignments between actual spot nodes and fitted spot nodes (where $\left( i,j \right)$ fall within the original $m \times n$ block) are retained as valid matches, while any assignment involving an auxiliary node implies that the corresponding spot is effectively unmatched or rejected as an outlier.

# Supplementary Note 2: Detailed Atom Search Optimization (ASO) algorithm

As stated in the " Materials and methods: Global matching strategy" section of the main text, we employ the Atom Search Optimization (ASO) algorithm^3^ to iteratively update the Zernike coefficients $\{C_{1},C_{2},\ldots,C_{15}\}$ to minimize the global matching cost $\mathcal{L}$ defined in Equation (5) of the main text. ASO is a physics-inspired meta-heuristic optimization algorithm that mathematically models the motion of atoms in a molecular system, where the interaction forces (attraction and repulsion) and constraint forces govern the movement of atoms toward a stable state (global minimum).

1. Initialization

Let $N$ be the population size of the atoms (candidate wavefronts). The position of the $i$-th atom at iteration $t$, denoted as $X_{i}\left( t \right)=\left[ C_{i,1},C_{i,2},\ldots,C_{i,15} \right]$, represents a specific set of Zernike coefficients. For the first frame or when no prior is available, the positions are randomly initialized within a bounded range defined by the physical limits of the SHWS. For continuous wavefront measurement, as mentioned in the main text, the converged coefficients from the previous frame are used to initialize the population for the current frame to accelerate convergence.

2. Fitness Evaluation and Mass Calculation

At each iteration $t$, the fitness of the $i$-th atom is evaluated by the matching cost function:

$$\begin{aligned} Fit_{i}\left( t \right)\mathcal{=L}\left( X_{i}\left( t \right) \right)\#\left( 5 \right) \end{aligned}$$

where $\mathcal{L}$ is the minimum total Euclidean distance calculated by the Jonker-Volgenant algorithm. The mass $m_{i}\left( t \right)$ of the $i$-th atom acts as a descriptor of its solution quality; atoms with better fitness (lower matching cost) have larger inertia. The mass is calculated as:

$$\begin{aligned} m_{i}\left( t \right)=\frac{M_{i}\left( t \right)}{\sum_{j=1}^{N} M_{j}\left( t \right)}\#\left( 6 \right) \end{aligned}$$

$$\begin{aligned} M_{i}\left( t \right)=e^{-\frac{Fit_{i}\left( t \right)-Fit_{best}\left( t \right)}{Fit_{worst}\left( t \right)-Fit_{best}\left( t \right)}}\#\left( 7 \right) \end{aligned}$$

where $Fit_{best}\left( t \right)$ and $Fit_{worst}\left( t \right)$ are the minimum and maximum matching costs in the current population, respectively.

3. Interaction Force and Acceleration

The motion of the $i$-th atom is driven by the interaction force $F_{i}$ from other atoms and the constraint force $G_{i}$ from the best atom. The interaction force acting on the $i$-th atom from the $j$-th atom in the $d$-th dimension is derived from the Lennard-Jones potential:

$$\begin{aligned} F_{ij}^{d}\left( t \right)=-\eta\left( t \right)\left[ 2\left( h_{ij}\left( t \right) \right)^{-13}-\left( h_{ij}\left( t \right) \right)^{-7} \right]\#\left( 8 \right) \end{aligned}$$

where $\eta\left( t \right)$ is a depth function adjusting the repulsion/attraction regions, and $h_{ij}$ is related to the distance between atoms:

$$\begin{aligned} h_{ij}\left( t \right)={\parallel X_{i}\left( t \right)-X_{j}\left( t \right)\parallel}_{2}+\delta\#\left( 9 \right) \end{aligned}$$

with a small constant $\delta={10}^{-6}$ to avoid singularity (e.g., division by zero). The total acceleration $a_{i}^{d}\left( t \right)$ of the $i$-th atom in the $d$-th dimension (corresponding to the $d$-th Zernike coefficient) is calculated as:

$$\begin{aligned} a_{i}^{d}\left( t \right)=\alpha\sum_{j\in K_{best}} \frac{rand_{j}F_{ij}^{d}\left( t \right)}{m_{i}\left( t \right)}+\beta\left( X_{best}^{d}\left( t \right)-X_{i}^{d}\left( t \right) \right)\#\left( 10 \right) \end{aligned}$$

Here, the first term represents the random interaction from the $K_{best}$ neighbors (promoting global search), and the second term represents the gravitational pull from the global best atom $X_{best}$ (promoting local convergence). $\alpha$ and $\beta$ are weighting factors.

4. Position Update

Finally, the velocity $v_{i}^{d}$ and position $X_{i}^{d}$ (Zernike coefficient value) are updated as follows:

$$\begin{aligned} v_{i}^{d}\left( t+1 \right)=rand_{i}\cdot v_{i}^{d}\left( t \right)+a_{i}^{d}\left( t \right)\#\left( 11 \right) \end{aligned}$$

$$\begin{aligned} X_{i}^{d}\left( t+1 \right)=X_{i}^{d}\left( t \right)+v_{i}^{d}\left( t+1 \right)\#\left( 12 \right) \end{aligned}$$

The algorithm iterates these steps until the matching cost $\mathcal{L}$ falls below a preset threshold $\epsilon$ or the maximum number of iterations is reached.

# Reference

1 Jonker, R. & Volgenant, A. A shortest augmenting path algorithm for dense and sparse linear assignment problems. *Computing* **38**, 325–340 (1987).

2 Samiei, A. & Sun, L. Distributed Matching-By-Clone Hungarian-Based Algorithm for Task Allocation of Multiagent Systems. *IEEE Transactions on Robotics* **40**, 851–863 (2024).

3 Zhao, W., Wang, L. & Zhang, Z. Atom search optimization and its application to solve a hydrogeologic parameter estimation problem. *Knowledge-Based Systems* **163**, 283–304 (2019).
